# Impacts of Cues on Learning and Attention in Immersive 360-Degree Video: An Eye-Tracking Study

**DOI:** 10.3389/fpsyg.2021.792069

**Published:** 2022-01-27

**Authors:** Rui Liu, Xiang Xu, Hairu Yang, Zhenhua Li, Guan Huang

**Affiliations:** Department of Educational Technology, Institute of Education, China West Normal University, Nanchong, China

**Keywords:** immersive 360-degree video, signaling, cues, learning outcome, attention allocation, eye-tracking technologies

## Abstract

Immersive 360-degree video has become a new learning resource because of its immersive sensory experience. This study examined the effects of textual and visual cues on learning and attention in immersive 360-degree video by using eye-tracking equipment integrated in a virtual reality head-mounted display. Participants (n = 110) were randomly assigned to one of four conditions: (1) no cues, (2) textual cues in the initial field of view (FOV), (3) textual cues outside the initial FOV, and (4) textual cues outside the initial FOV + visual cues. The results showed that the cues (annotations or annotations + arrows) helped learners achieve better learning outcomes and spend more time focusing on the areas with cues. In addition, the study found a serious imbalance in the distribution of learners’ attention in each region of the video. The attention directed to textual cues in the initial FOV is much higher than the attention directed to textual cues outside the initial FOV. Adding visual cues can effectively direct attention to textual cues outside the initial FOV and alleviate the imbalance of attention distribution. Consequently, adding cues to immersive 360-degree video can be an appropriate approach to promote learning and guide attention in immersive 360-degree video learning environments. This study provided new insights into the design and development of immersive 360-degree video instructional resources.

## Introduction

Virtual reality (VR) can create an immersive three-dimensional interactive virtual environment. As a new learning tool, VR is increasingly used in education (Radianti et al., 2020). Immersive 360-degree video, which is a new type of video based on VR technology, possesses great application potential in education. Immersive 360-degree video differs very much from traditional video with regard to experience. Currently, there are few relevant studies on the impact of these differences on cognition. The Cognitive Theory of Multimedia Learning (CTML) divides multimedia learning materials into words and pictures. People process information *via* visual/pictorial and auditory/verbal channels (Mayer, 2005). As in other VR environments, the presentation of information in immersive 360-degree video is (as in traditional multimedia) mainly through spoken words and animation (Parong and Mayer, 2018). Therefore, the CTML may provide a theoretical basis for us to study cognition in an immersive 360-degree video learning environment. In a VR learning environment, due to the increase in visual range and interactivity, information capacity is greatly improved, thus possibly causing distraction, increasing unnecessary cognitive load, and reducing the learning effect (Parong and Mayer, 2020). Additionally, in immersive 360-degree video, learners’ field of view (FOV) has limitations. Learners can observe only partial pictures of the video at a certain point in time; consequently, learners may miss important learning content. According to the signal principle of CTML, adding cues to multimedia learning materials is a means to effectively guide learners’ attention, improve learning efficiency and reduce cognitive load in learning (Karich et al., 2014). Then, can adding cues to immersive 360-degree video reduce the interference of irrelevant processing on learning, effectively guide attention, and promote learning? Therefore, the aim of this study was to investigate the effect of cues on learning and attention in an immersive 360-degree video learning environment.

### Virtual Reality and Immersive 360-Degree Video

Virtual reality uses computer technology to simulate real-time interactions between 3D entities in the virtual world so that participants can immerse themselves in a pseudonatural way by perceiving the motion channel. There are many advantages to using VR in a teaching or training environment. For example, VR can simulate the use of rare and expensive tools, reduce learning risks and costs, simulate complex or dangerous situations, control the learning environment or situation, and reproduce elements of real life (Arnaldi et al., 2018). Some studies have shown that VR technology can improve students’ learning motivation and learning achievements (Gunn et al., 2017; Kavanagh et al., 2017; Makransky et al., 2019).

Although VR has been proven to be helpful for learning, the technical and financial cost of interactive 3D VR resource development is high, thus greatly hindering the popularization of virtual reality in teaching (Yang et al., 2010). The emergence of immersive 360-degree video provides a good solution. Panoramic or 360-degree video is a new type of video in which users can adjust the viewing direction at will. According to different viewing methods, 360-degree video can be divided into non-immersive 360-degree video and immersive 360-degree video (also known as 360-degree VR video). Immersive 360-degree video needs to be played on special VR head-mounted displays (HMDs), such as the HTC Vive or Oculus Rift. With the help of simple tools, such as cardboard, smartphones can also be converted into simple VR headsets (Rupp et al., 2019). Compared with the development cost of 3D interactive VR resources, the development cost of immersive 360-degree video is lower. After simple training, teachers can develop their own learning content (Chien et al., 2020).

The CTML holds that when learners take the initiative in cognitive processing, the best learning effect is produced (Mayer, 2005). An immersive virtual learning environment can help learners establish the connection between new knowledge and existing knowledge; encourage learners to actively select, organize and integrate information; and achieve meaningful learning (Araiza-Alba et al., 2021). A previous study confirmed that compared with 360-degree video that is watched directly on the screen, immersive 360-degree video can provide greater immersion, a more positive learning experience and a better learning effect (Rupp et al., 2019). Other studies have shown that the highly immersive user experience of immersive 360-degree video can activate the sense of presence and enhance learning interest and engagement (Rupp et al., 2016; Harrington et al., 2018).

Compared with traditional video, 360-degree video provides users with a larger visual range. In the VR environment, although users can adjust the viewing angle by turning their head to focus on content outside the current FOV, the content observed at a certain time is limited (Zhu et al., 2018). A limited view means that it is easy to overlook important content when watching immersive 360-degree video (Ardisara and Fung, 2018). The Cognitive Load Theory (CLT) holds that human working memory capacity is limited. Any learning task consumes cognitive resources and produces cognitive load (Sweller, 1988). A free visual angle and redundant visual information may produce a higher cognitive load, thereby resulting in cognitive overload. Therefore, when watching immersive 360-degree videos, the attention allocation of learners may differ from their attention when they watch traditional videos, and excessive cognitive load may affect learning.

### Signal Principle in Multimedia Learning

The CTML assumes that humans process information *via* two channels: auditory/verbal and visual/pictorial. The information processing capacity of each channel is limited. Humans are active agents who process cognitive resources and carry out meaningful learning through selection, organization and integration (Mayer, 2014). Based on the above assumptions and a series of empirical studies, Mayer (2014) proposed multimedia design principles that provided a basis for designing an effective multimedia learning environment. Among these principles, the signaling principle (or cueing principle) suggests that the use of cues in learning materials to guide learners’ attention to relevant information or highlight key content will produce a better learning effect (Gog, 2014). Since not all learning situations involve teachers who monitor learning progress, it is necessary to use attention-guiding features in learning materials to coordinate the selection of relevant information (Schneider et al., 2018). In addition to performing a guiding function, cues can emphasize the topics and organization of instruction and make the relationship between elements more salient to promote their integration (De Koning et al., 2009). In a VR learning environment, because the presentation of visual stimuli is converted from the 2D plane to 360-degree all-around visibility, the search and orientation processes of learning materials may become more complicated (Albus et al., 2021). Thus, in immersive 360-degree videos, cues can be used to help learners understand the relationships between information, reduce unnecessary visual searches, and enhance auditory narration.

Cues in multimedia learning can be divided into textual cues and visual cues (Mayer, 2021). Textual cues include headings, annotations, summaries, font colors, text picture references, and intonation (Schneider et al., 2018). Annotations are common textual cues that can highlight the internal relationships between information, support mapping and integration processes, and repeat the crucial terms of the auditory text as needed to help deepen learners’ understanding of information (Vogt et al., 2021). A study found that in VR, annotations could improve learners’ recall performance and germane cognitive load (Albus et al., 2021). In immersive 360-degree videos, visual information is rich and intense, while narrative information acquired through the auditory channel is transient and easily overlooked. Based on the spatial and temporal contiguity principles (Mayer, 2005), annotations can be placed next to key pictures as a supplement and emphasis, and appear simultaneously with the narration, thus helping learners establish a mapping relationship between the pictures and the narration and promoting the further organization and integration of information.

Visual cues include arrows, colors, gestures, flashes, labels, and graphic organizers, all of which can guide learners to pay attention to key information (Schneider et al., 2018). Mayer (2017) found that in multimedia learning, if important content is indicated by highlights, colors or arrows, learners’ performance can be improved. The FOV is the size of the visual field in the degrees of the visual angle that can be viewed instantaneously (Bowman and McMahan, 2007). A previous study suggested that in a VR environment, the FOV affects enjoyment, memory, and simulator sickness (Lin et al., 2002). Although the field of regard (FOR) of immersive 360-degree video is large (close to the real environment), the observer’s FOV is limited and even smaller than in the real environment (Jang et al., 2016; Miola et al., 2021). Therefore, it is necessary to constantly move the head or body while watching to perceive information beyond the current FOV. A study suggested that in a VR environment, in terms of attention to the target stimuli, although the detection time in the FOV is faster than that outside the FOV, the time difference between the two is significantly shortened if cues are added (Jang et al., 2016). Therefore, visual cues may play a positive role in directing and locating attention in immersive 360-degree videos.

The eye-mind hypothesis states that learners’ fixation on certain information and psychological processing of the information are carried out at the same time; that is, the information currently being viewed by human eyes is the information currently being processed by the human brain (Just and Carpenter, 1976). Therefore, eye movement data can provide effective information about learners’ cognitive processing (Ballard et al., 1997). The influence of cues may stem from guiding attention to relevant information (Lorch, 1989). Since eye movement measurements are often used to reveal visual attention on the items in the scene and changes in the focus of visual attention (Just and Carpenter, 1980), eye-tracking technology can be used to reveal the influence of cues on learners’ attention. Some eye-tracking studies found that visual cues can guide learners’ attention, enhance visual search, effectively improve learning speed and reduce the interference of extraneous cognitive load (Tabbers et al., 2008; De Koning et al., 2010; Ozcelik et al., 2010; Kuhl et al., 2012). The study of eye movement behavior in VR environments is a new research field. A study used eye movement technology to predict the movement path of the eyes and head in 360-degree videos and explored the relationship between social anxiety and attention (Rubin et al., 2020). However, there are few eye-tracking studies on the impact of cues on attention in virtual reality environments. Therefore, this study used eye-tracking equipment integrated into a VR HMD to evaluate learning behaviors in an immersive 360-degree video learning environment.

### The Present Study

The main aim of this study was to examine the effects of cues in immersive 360-degree video on learners’ learning outcomes and attention allocation. It was assumed that learning materials applying the signal principle could contribute to attention allocation and lead to higher learning outcomes. Based on existing studies, we proposed the following research questions and hypotheses:

Q1: In an immersive 360-degree video learning environment, does the addition of textual cues affect learning outcomes and attention allocation?

H1: Compared to learners studying without textual cues, learners studying immersive 360-degree video with textual cues were expected to have better learning outcomes (H1a) and a longer fixation duration (H1b). The rationale for this prediction is that textual cues help guide attention to relevant information and timely repetition of the narrative content (the Signaling Principle: Mayer, 2021). Moreover, learners in the cues condition may attend to signaled elements more frequently (Scheiter and Eitel, 2015; Wang et al., 2020).

Q2: Do textual cues have different effects on learning outcomes and attention allocation when they are inside or outside the learner’s initial FOV?

H2: Learners were expected to perform better (H2a) and to fixate more on the annotated areas (H2b) when textual cues were inside rather than outside the initial FOV. The rationale for this prediction is that students learn better when textual cues and relevant information are presented close to each other rather than separately (the spatial contiguity principle: Mayer, 2021).

Q3: When the textual cues are outside the initial FOV, does the presence or absence of visual cues have different effects on learning outcomes and attention allocation?

H3: Compared to learners studying without textual cues, learners studying immersive 360-degree video with directional visual cues were expected to have better learning outcomes (H3a). Moreover, learners were expected to fixate more on the annotated areas that were guided by visual cues (H3b). When learners’ cognitive resources are consumed by excessive visual searching, learning will be hindered if learners are not guided by appropriate cues (Ozcelik et al., 2010).

## Materials and Methods

### Participants and Design

We recruited 112 undergraduates from a university in China. Two participants had to be excluded from data analyses due to technical issues with eye-tracking device. Of the remaining 110 participants, 74 were females (*M*_age_ = 20.52, *SD*_age_ = 1.30, age range: 18–24). More than 95% of participants had no experience with virtual reality, and all of the participants had no VR or 360-degree video learning experience. All participants had normal hearing and normal or corrected-to-normal vision. At the end of the study, the participants received a small gift (consisting of a notebook, pen and candy).

**FIGURE 1:**
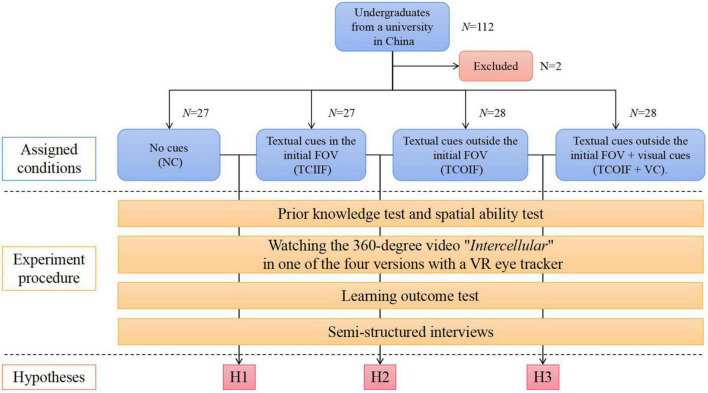
The experimental conditions and procedure.

This study adopted the mixed research method of experimental research and semi-structured interviews. We used a single factor intergroup design with 4 groups. The participants were randomly assigned to one of four conditions by simple random sampling: the no cues (NC) group (*n* = 27), the textual cues in the initial FOV (TCIIF) group (*n* = 27), the textual cues outside the initial FOV (TCOIF) group (*n* = 28), and the textual cues outside the initial FOV + visual cues (TCOIF + VC) group (*n* = 28). Concerning dependent variables, we measured the learning outcomes of the participants and main eye movement indicators (total fixation duration, fixation duration on annotation areas of interest (AOIs), fixation duration on initial FOV AOIs and fixation heatmaps). Concerning control variables, we considered prior knowledge and spatial ability. In addition, semi-structured interviews were conducted with the participants after the experiment. The experimental conditions and procedure are shown in Figure 1.

### Devices and Materials

#### Virtual Reality and Eye-Tracking Devices

An HTC Vive Pro HMD was used as a display device with a resolution of 2160 × 1200 (1080 × 1200 for each eye) and a refresh rate of 90 Hz. The HMD binocular FOV was 110°. The Tobii Pro VR eye tracker was used as eye-tracking equipment. The device was integrated into the HTC Vive Pro HMD with an accuracy of 0.5° and a sampling frequency of 120 Hz. Tobii Pro lab 1.1 software was used to perform calibration, play videos, and analyze the eye-tracking data after the experiment.

#### Learning Materials

The 360-degree video “*Intercellular*,” developed by Random42 Scientific Communication, was used as the learning material. The video presented and explained the forms and functions of various cells in the human body by using realistic 3D animation. The total video duration is 3 min and 24 s, and the resolution is 2,304 × 1,080. The original video was provided with English narration, with English annotations at key positions and no subtitles. To reduce the interference of irrelevant factors, we converted the video into Chinese narrations and annotations and invited domain-related experts to improve the translation to ensure accuracy and fluency.

**FIGURE 2:**
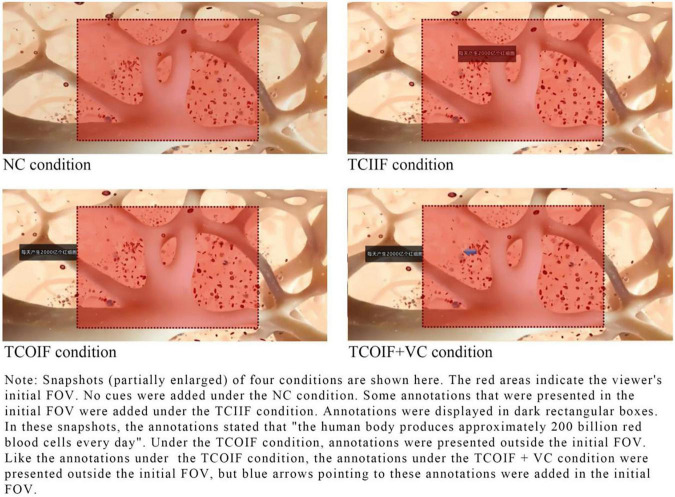
Snapshots of the four conditions.

According to the four conditions of the experimental design, the video was processed into four corresponding versions. Figure 2 provides snapshots of the four conditions: (1) No cues (NC), i.e., there were no textual or visual cues in the learning materials. (2) Textual cues in the initial FOV (TCIIF), in which key information was presented in the learner’s initial FOV in the form of annotations. We added 17 annotations. Some annotations were explanations of the pictures; for example, the annotation “red blood cells” was added next to red blood cells when they were presented. Other annotations were repetitions of the narration; the annotation “the human body produces approximately 200 billion red blood cells every day” was used. (3) Textual cues outside the initial FOV (TCOIF). As with the TCIIF condition, key information was presented in the form of annotations. The difference was that in the TCOIF condition, most of the annotations appeared outside the learners’ initial FOV. That is, learners needed to turn their heads to see these annotations. (4) Textual cues outside the initial FOV + visual cues (TCOIF + VC). As with the TCOIF condition, the key information was presented outside the learners’ initial FOV in the form of annotations. The difference was that in the TCOIF + VC condition, arrows pointing to these annotations were added in the initial FOV.

### Measures

A prior knowledge test was used to assess the level of biological knowledge related to the learning task. The questionnaire consisted of five short self-evaluation questions, such as “I can describe the main structure of cells” and “I can explain the cause of leukemia.” From the five options, participants were asked to choose the option that fit their actual situation. The options ranged from “completely inconsistent” to “completely consistent” and corresponded consecutively to 1–5 points. The prior knowledge test showed high internal consistency (α = 0.81).

The paper-folding test and the card rotation test were used to measure the participants’ spatial ability (Ekstrom et al., 1976). The two tests assessed two types of spatial abilities—spatial visualization and mental rotation. The participants were given one point for each question they answered correctly, with the final total indicating each participant’s spatial ability.

The learning outcome test consisted of 11 questions, all closely related to the learning material and developed by two domain-related experts. The test was divided into multiple-choice and single-choice questions. Each question was worth 1 point for a total score of 11 points. The answers to all the questions were presented in the video narration. Considering that the differences among the four conditions were reflected mainly in the cues, the answers to most of the questions were emphasized in the annotations of the video (except for the NC group). An example of a single-choice question is “What organizational structure is shown in the figure? A. synapses; B. leukocytes; C. immune cells; D. axons.” An example of a multiple-choice question is “Which structures in the gut can assist in food digestion and nutrient absorption? A. villi; B. bacterial colony; C. capillaries; D. microvilli.” The spatial ability test showed moderate internal consistency (α = 0.71).

Eye movement indexes, such as the total fixation duration of the learning materials, the total fixation duration of the AOIs, and fixation heatmaps, were used. The total fixation duration was the total amount of time of all the fixation durations in specified AOIs; this index can be used to reflect the processing depth or degree of attention of learners to the content (Ponce and Mayer, 2014). The heatmaps used different colors to illustrate the participants’ fixation duration in the stimulation area; the heatmaps reflected the participants’ overall allocation of cognitive resources (Wang et al., 2020). Red usually indicates the longest fixation duration, and green indicates the shortest fixation duration. There are different levels between the two colors.

A previous study suggested that the combination of a concurrent verbal protocol and eye movement data analysis can enhance insight into cognition (Gog et al., 2010). To better analyze the cognition reflected by eye movement data and to understand learners’ subjective feelings, semi-structured interviews were conducted after the experiment. During the interviews, the participants answered several open-ended questions, such as, “Do you think immersive 360-degree video is helpful for learning? What do you think are the advantages and disadvantages of immersive 360-degree video? Do the annotations and arrows in the video help clarify the learning content?”

### Procedure

First, the participants entered the preparation room, provided basic information, and took a test of prior knowledge and spatial ability. Next, the participants were brought into the testing room and seated. The participants read the instructions of the experiment to understand the experiment content, and the experiment assistant taught the participants how to use the VR HMD. Then, the participants wore the HTC Vive Pro HMD, performed five-point calibration of the eye movement system, and watched video materials randomly assigned under one of the four conditions. After watching the video, the participants were tested for learning outcomes and interviewed. The entire process took approximately 15 min.

## Results

### Descriptive Statistics (Means and Standard Deviations)

Table 1 shows the descriptive data statistics of prior knowledge, spatial ability, learning outcomes, and main eye movement indicators. We conducted an ANOVA with regard to prior knowledge and spatial ability. The results showed no significant differences among the groups in their prior knowledge [*F*(3, 106) = 0.44, *p* = 0.724)] and spatial ability [*F*(3, 106) = 1.51, *p* = 0.216)].

**TABLE 1:** Descriptive data for all variables under the four conditions.

Dependent variables	NC group (*N* = 27)		TCIIF group (*N* = 27)		TCOIF group (*N* = 28)		TCOIF + VC group (*N* = 28)	
	*M*	*SD*	*M*	*SD*	*M*	*SD*	*M*	*SD*
Prior knowledge	16.44	3.80	15.41	3.58	16.36	4.25	16.07	2.99
Spatial ability	9.63	2.29	10.70	2.16	10.04	2.30	9.61	1.93
Learning outcome	5.19	1.86	7.44	1.22	6.21	1.79	7.14	1.80
Total fixation duration (in seconds)	126.78	28.40	139.33	17.25	130.45	20.64	123.19	19.79
Fixation duration on annotation AOIs (in seconds)	N/A	N/A	14.03	5.60	6.98	3.86	13.64	6.06
Fixation duration on initial FOV AOIs (in seconds)	123.99	29.07	138.08	18.61	127.99	21.65	116.05	20.53

*The maximum score on the prior knowledge test was 25; the maximum score on the spatial ability test was 15; and the maximum score on the learning outcome test was 11.*

### Learning Outcomes

The descriptive results showed that the TCIIF group had the best learning performance, followed by the learning performance of the TCOIF + VC group. The NC group had the worst learning performance (see Table 1). We used one-factor ANOVA to analyze the differences in learning outcomes between the four conditions. The results showed that there were significant differences in the learning outcomes of participants between the different conditions, i.e., *F*(3, 106) = 9.87, *p* < 0.001. The follow-up *post hoc* analysis using a least significant difference (LSD) test showed that the TCIIF group (*p* < 0.001) (H1a), the TCOIF group (*p* < 0.05) and the TCOIF + VC group (*p* < 0.001) outperformed the NC group, and the TCIIF group outperformed the TCOIF group (*p* < 0.05) (H2a). There was no significant difference between the TCIIF group and the TCOIF + VC group (*p* > 0.05). These results were consistent with each hypothesis; that is, adding cues (annotations, arrows, or annotations + arrows) helped to improve learners’ learning effect, and the position of the annotations affected learning outcomes.

### Eye-Tracking Outcomes

#### Fixation Duration

To explore the effect of textual cues on learners’ attention allocation, one-factor ANOVA was used to analyze the total fixation duration under the four conditions. The results showed that there were significant differences in total fixation duration under the different conditions, i.e., *F*(3, 106) = 2.74, *p* = 0.047. The follow-up *post hoc* analysis using an LSD test showed that the total fixation duration in the TCIIF group was significantly longer than that in the NC group (*p* = 0.038) (H1b) and the TCOIF group (*p* = 0.007). There was no significant difference in the total fixation duration between the NC group, the TCOIF group and the TCOIF + VC group. The results showed that learners paid more attention to the learning material when there were textual cues in the initial FOV.

To explore whether the location of textual cues and the guidance of visual cues affected learners’ attention to textual cues, we set the annotation areas of the TCIIF group, the TCOIF group and the TCOIF + VC group as AOIs and conducted an ANOVA for the total fixation duration of the AOIs between the groups. The results showed that the total fixation duration of the annotation areas was significantly different under different conditions, *F*(2, 80) = 15.79, *p* < 0.001. The follow-up *post hoc* analysis using an LSD test showed that the total fixation duration of the annotation areas of the TCOIF group was significantly lower than that of the TCIIF group (*p* < 0.001) (H2b) and the TCOIF + VC group (*p* < 0.001) (H3b), but there was no significant difference between the TCIIF and TCOIF + VC groups (*p* > 0.05). The results were consistent with the hypotheses. Attention to annotations outside the initial FOV was much lower than that inside the initial FOV, and the arrows effectively guided learners’ attention to annotations outside the FOV.

To explore the effect of cueing on the allocation of attention in the initial FOV, we set areas of the initial FOV to AOIs and performed an ANOVA. The results showed that there was a significant difference in the total fixation duration of the initial FOV between groups, i.e., *F*(3, 106) = 4.44, *p* = 0.006. The follow-up *post hoc* analysis using an LSD test showed that the total fixation duration of the initial FOV in the NC group (*p* = 0.025) and the TCOIF + VC group (*p* = 0.001) was significantly shorter than that in the TCIIF group. There was no significant difference between the NC group and the TCOIF + VC group, and there was no significant difference between the TCOIF group and the other groups.

#### Heatmaps

**FIGURE 3:**
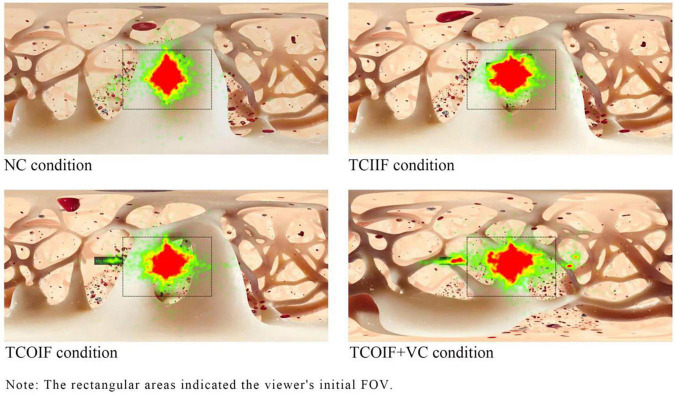
Fixation heatmaps of the four conditions.

The fixation heatmaps reflected the differences in learners’ fixation duration in different areas during the video playback time. As shown in Figure 3, under the four conditions, participants allocated most of their attention to the initial FOV (the rectangular areas in the figure are the learners’ initial FOV). The annotation areas outside the initial FOV were significantly hotter in the TCOIF + VC group than in the TCOIF group, thus indicating that these areas received more attention and suggesting that visual cues (arrows) had an obvious guiding effect on attention.

## Discussion

The main purpose of this study was to explore the effects of cues on learning outcomes and attention allocation when using immersive 360-degree video for learning. We assumed that the signal principle based on the CTML was also applicable to the immersive 360-degree video learning environment and assumed that whether the cues were in the initial FOV would have different effects on learning and attention. The experimental results supported our hypothesis that in immersive 360-degree videos, cues guide attention and help improve learners’ learning performance. Additionally, there was a serious imbalance in learners’ attention allocation in each area of the video pictures, and the addition of visual cues affected attention allocation in each area. According to the interview results, the participants believed that an overly strong immersion would distract their attention, while a limited FOV might cause them to miss important information, and a lack of VR experience would also hinder learning. Some participants mentioned that the arrows helped them locate key information more quickly.

### Effects of Annotations on Learning Outcomes and Attention Allocation

To answer the first question, we analyzed whether learning outcomes (H1a) and attention (H1b) within the initial FOV were affected by annotations. We found that participants who watched annotated learning materials outperformed the control group without annotations. Consistent with our expectations, in the immersive 360-degree video, adding annotations positively affected cognition, thereby supporting a previous study (Albus et al., 2021). This finding has two possible explanations. First, when annotations and narration appear at the same time, learners more easily associate auditory and visual information; thus, using annotations and narration simultaneously can reduce unnecessary visual searches (Jeung et al., 1997). According to the CLT, reducing unnecessary visual search processes can reduce extraneous cognitive load. Learners will have more cognitive resources for learning and can understand learning materials more easily (Sweller et al., 1998). Second, due to the simultaneous presentation of annotations and animations, the interconnection between verbal and non-verbal systems was enhanced to achieve more efficient dual coding (the Multimedia Principle: Mayer, 2021). The eye-movement data also matched expectations: the presence of annotations led to a longer fixation duration; this finding is consistent with that of a previous study (De Koning et al., 2007; Wang et al., 2020). Some participants mentioned that the learning materials were extremely immersive and stimulating, and the large visual range and fast-paced animation made the participants lose focus on the learning objectives. However, when annotations were present, learners’ attention was attracted by them, thus reducing unnecessary visual searches and allowing learners to focus more on key content.

### Effects of Annotation Position on Learning Outcomes and Attention Allocation

To answer the second question, we analyzed the learning outcomes and attention allocation of the annotations inside and outside the initial FOV. We found that compared with learners in the TCOIF group, learners in the TCIIF group achieved better learning scores (H2a). This finding has two possible explanations. First, for immersive 360-degree video, although an immersive scene with an almost 360-degree field of view is created, there is still a main FOV that is the initial default FOV when watching, and most of the key content is displayed in this FOV. According to the spatial contiguity principle (Mayer, 2021), when annotations are within the initial FOV, learners can better associate annotations with pictures without consuming cognitive resources in the visual search. In this way, it is more likely that learners will retain the information in their short-term memory (Mayer, 2021). Second, when the annotations were outside the initial FOV, if the learner did not turn his or her head to change the FOV, they would be ignored entirely. This was completely different from the situation of classical multimedia learning. Many participants said that the blind area due to the large range of vision caused them to miss important details. The results of eye movement records also supported this. Compared with learners in the TCOIF group, learners in the TCIIF group spent a greater fixation duration on the annotation areas (H2b). More interestingly, up to 57% of participants in the TCOIF group had zero fixation duration for the annotations outside the initial FOV. Therefore, for these participants, the annotations outside the initial FOV did not affect the participants’ cognition. The heatmaps also showed that most of the participants’ attention was focused on the initial FOV. We suspect that this phenomenon may be related to the participants’ VR experience. Some studies have supported that prior experience using the applied VR technologies is effective for reducing simulator sickness and improving behaviors in VR (Shafer et al., 2017; Mittelstaedt et al., 2018). Most of the participants in this study had no experience watching immersive 360-degree video. Although all participants were informed in advance that they could see more content by turning their heads, during the formal experience, due to the high immersion and fast-paced sensory stimulation, some participants had no time or forgot to turn their heads but subconsciously viewed the 360-degree video as a traditional video. In the interviews, the participants also mentioned that the lack of VR experience negatively affected the perception of visual blind areas. However, considering the poor boundary vision of the human visual system (Johnson, 2021), if the position of the annotations is not in the center of the FOV, even if participants turn their heads, participants are likely to miss the annotations due to the interference of the fast-moving picture. Therefore, if annotations (or other key content items) appear outside the initial FOV, it is necessary to use some means to guide learners’ attention and the FOV.

### Effects of Visual Cues on Learning Outcomes and Attention Allocation

Our third question explored the effects of visual cues on learning outcomes and attention allocation. When adding annotations outside the initial FOV, the additional visual cues (arrows) more positively affected the learning outcomes (H3a). Additionally, the eye movement results showed that visual cues had an obvious guiding effect on learners’ attention: the TCOIF + VC group spent more fixation time in the annotation area (H3b). Previous eye-tracking studies have found that learners pay more attention to relevant areas when guided by visual cues (Jamet, 2014; Wang et al., 2020); this finding is consistent with our results. In contrast to previous studies, we examined the guiding effect of visual cues on the FOV in immersive 360-degree video. We found that compared with the TCOIF group, the TCOIF + VC group showed a significant increase (from 43 to 96%) in the proportion of attention to annotations outside the initial FOV. The CTML holds that meaningful learning includes three basic cognitive processes, namely, selection, organization and integration, and the selection of key information precedes other processes of learning (Alpizar et al., 2020). In the immersive 360-degree video learning environment, because learners’ FOV accounts for only a small part of the visual range, the difficulty of information selection is significantly higher than when using traditional video. Therefore, it is particularly important to use visual cues to guide learners’ attention, especially to the information outside the initial FOV. Some participants mentioned that arrows could help them consciously notice annotations outside their FOV. According to the limited capacity assumption, the information that people can process at one time is very limited (Mayer, 2005). When visual cues lead learners’ attention to annotations (or other AOIs), learners will inevitably reduce their visual attention to other pictures. Eye movement data analysis also confirmed that compared with learners in the TCIIF group, learners in the TCOIF + VC group had significantly less fixation duration in the initial FOV. The positioning assistance function of visual cues can reduce the difficulty of spatial positioning in an immersive 360-degree video learning environment. However, visual cues also consume limited cognitive resources and frequently intervene in the allocation of attention through exogenous positioning, which may increase irrelevant processing and lead to cognitive overload. Therefore, the reasonable and appropriate use of visual cues may be the key to the success of immersive 360-degree video instructional design.

### Limitations and Suggestions

This study has several limitations. First, this study involved short-term learning provided ifdn a lab setting. Although this study provided preliminary empirical results on the effectiveness of adding cues to immersive 360-degree video learning materials, the external validity of the results needs to be tested in authentic learning contexts. Second, we did not measure the effect of the learner’s VR experience. Almost all participants in this study had no experience in immersive VR learning. Although, in the interviews, some participants mentioned that immersive VR devices were not friendly to novices and required some time to learn and adapt, the impact caused by lack of experience needs further research. Third, there were certain restrictions on learning materials. The learning materials used in this study were mainly declarative knowledge, and the length was only 3 min. Therefore, measuring learning outcomes involved primarily assessing the retention of knowledge and not the transfer of knowledge. Furthermore, the type of learning materials might also affect the experimental results. For example, compared with the form of 3D animation adopted in this study, 360-degree videos shot based on real scenes may be more prone to place illusions because these scenes are closer to the real world. Place illusion can improve performance within VR by providing accurate perceptual cues to users (Slater and Sanchez-Vives, 2016). Fourth, we evaluated the impact of only annotations and arrows and did not involve other features of textual and visual cues. Finally, this study controlled prior knowledge and spatial ability as interference variables and did not discuss the interaction between learners’ individual characteristics and cues, such as prior knowledge and spatial ability.

Based on the current research findings, we propose the following for relevant studies in the future:

(1) Future work should involve investigating more authentic learning contexts.
(2) We suggest examining the effect of learners’ VR experience on attentional tendency.
(3) We suggest using more complex learning materials to evaluate learning outcomes at the level of other cognitive learning objectives.
(4) We recommend evaluating the effects of different types of learning materials, such as 360-degree video of real scenes.
(5) We suggest that future studies investigate the effects of other cue features, such as vocal emphasis, colors, flashing, direction of cues, number of cues, and existence time of cues.
(6) The interaction between learners’ individual characteristics (prior knowledge, spatial ability, cognitive style, age and motivation, etc.) and cues is also an interesting research direction, which is suggested to be investigated in future work.

## Conclusion

In this study, we explored the effects of cues on learning outcomes and attention allocation in an immersive 360-degree video learning environment. In comparison to previous studies, we focused on finding patterns of attention distribution by using eye-tracking studies (Rupp et al., 2016; Albus et al., 2021; Vogt et al., 2021). The results showed that textual and visual cues positively affected cognition, thereby proving that the signal principle of CTML is also applicable in the immersive 360-degree video learning environment. Additionally, we found that learners paid much more attention to the initial FOV than other regions, thus leading learners to miss most of the information outside the initial FOV. This result might be related to the large range of vision and the lack of VR experience. In view of this phenomenon, we found that using visual cues to guide attention to blind areas of sight was an effective solution. Therefore, we suggest supporting learners with cues when designing immersive 360-degree video learning environments. In short, the results of this study not only expand the applicable scenarios of multimedia learning theory but also make a practical contribution to the rational design of immersive 360-degree video learning environments. In this study, we adopted eye-tracking technology, which provides a new idea and method for studying VR learning environments.

## Data Availability Statement

The raw data supporting the conclusions of this article will be made available by the authors, without undue reservation.

## Ethics Statement

The studies involving human participants were reviewed and approved by the Ethics Committee of China West Normal University. The patients/participants provided their written informed consent to participate in this study.

## Author Contributions

RL: writing—review and editing, methodology, manuscript revision, and funding acquisition. XX: acquisition of the original data, software, and formal analysis. HY: conceptualization and methodology. ZL and GH: reference management and critical manuscript revision. All authors contributed to the article and approved the submitted version.

## Conflict of Interest

The authors declare that the research was conducted in the absence of any commercial or financial relationships that could be construed as a potential conflict of interest.

## Publisher’s Note

All claims expressed in this article are solely those of the authors and do not necessarily represent those of their affiliated organizations, or those of the publisher, the editors and the reviewers. Any product that may be evaluated in this article, or claim that may be made by its manufacturer, is not guaranteed or endorsed by the publisher.
